# Guardians of the data: NER and LLMs for effective medical record anonymization in Brazilian Portuguese

**DOI:** 10.3389/fpubh.2025.1717303

**Published:** 2026-01-05

**Authors:** Mauricio Schiezaro, Guilherme Rosa, Bruno Augusto Goulart Campos, Helio Pedrini

**Affiliations:** 1 Venturus - Innovation and Technology, Campinas, Brazil; 2 School of Medical Sciences, UNICAMP, Campinas, Brazil; 3 Institute of Computing, UNICAMP, Campinas, Brazil

**Keywords:** generative models, large language models, medical data anonymization, named entity recognition, seq2seq, transformer models

## Abstract

**Introduction:**

The anonymization of medical records is essential to protect patient privacy while enabling the use of clinical data for research and Natural Language Processing (NLP) applications. However, for Brazilian Portuguese, the lack of publicly available and high-quality anonymized datasets limits progress in this area.

**Methods:**

In this study, we present *AnonyMed-BR*, a novel dataset of Brazilian medical records that includes both real and synthetic samples, manually annotated to identify personally identifiable information (PII) such as names, dates, locations, and healthcare identifiers. To benchmark our dataset and assess anonymization performance, we evaluate two anonymization strategies: (i) an extractive strategy based on Named Entity Recognition (NER) using BERT-based models, and (ii) a generative strategy using T5-based and GPT-based models to rewrite texts while masking sensitive entities. We conduct a comprehensive series of experiments to evaluate and compare anonymization strategies. Specifically, we assess the impact of incorporating synthetic generated records on model performance by contrasting models fine-tuned solely on real data with those fine-tuned on synthetic samples. We also investigate whether pre-training on biomedical corpora or task-specific fine-tuning more effectively improves performance in the anonymization task. Finally, to support robust evaluation, we introduce an *LLM-as-a-Judge* framework that leverages a reasoning Large Language Model (LLM) to score anonymization quality, estimate information loss, and assess reidentification risk. Model performance was primarily evaluated using the F1 score on a held-out test set.

**Results:**

All evaluated models achieved good performance in the anonymization task, with the best models reaching F1 scores above 0.90. Both extractive and generative approaches were effective in identifying and masking sensitive entities while preserving the clinical meaning of the texts. Experiments also revealed that including synthetic data improved model generalization, and that task-specific fine-tuning yielded greater performance gains than pre-training the model on biomedical domain.

**Discussion and conclusion:**

To the best of our knowledge, *AnonyMed-BR* is the first manually annotated anonymization dataset for Brazilian Portuguese medical texts, enabling systematic evaluation of both extractive and generative models. The dataset and methodology establish a foundation for privacy-preserving NLP research in the Brazilian healthcare context and the good performance achieved by all models demonstrates the feasibility of developing reliable anonymization systems for Brazilian clinical data. Importantly, the ability to anonymize sensitive information opens opportunities to create new datasets and train models for a variety of downstream tasks in the medical domain, such as clinical outcome prediction, medical entity recognition, diagnostic support, and patient stratification, fostering the growth of NLP research for Brazilian Portuguese healthcare texts. Motivated by our findings, future work includes a deeper exploration of synthetic data generation and utilization. Additionally, we plan to evaluate the models across different languages and textual domains, and to expand the dataset to cover these new languages and domains. These efforts aim to develop more complex anonymization systems with higher generalization capability, ultimately enabling broader applications and safer sharing of data in diverse research and operational settings. All resources are publicly available at https://github.com/venturusbr/AnonyMED-BR.

## Introduction

The growing demand for Natural Language Processing (NLP) models in the healthcare sector has highlighted the need for solutions adapted to languages other than English (1, 2). While significant progress has been made in English (3–6), the lack of datasets and models tailored to other languages, such as Brazilian Portuguese, presents a critical gap (7, 8). This scarcity makes it difficult to develop NLP tools across diverse linguistic and cultural contexts. This challenge is further amplified by data-privacy regulations such as the General Data Protection Regulation (GDPR) (9) and the Brazilian General Data Protection Law (LGPD) (10), which mandate the protection of personally identifiable information and sensitive health data in medical records.

Beyond legal compliance, ensuring the confidentiality of medical data is also crucial for preserving trust in the physician-patient relationship, which directly impacts the quality of care and patient adherence to treatment. Data breaches or unauthorized identification of sensitive information can cause significant harm to patients and damage the credibility of the institutions involved (11).

Before the adoption of NLP-based systems, anonymization of medical text relied heavily on rule-based and pattern-matching approaches, including handcrafted regular expressions, predefined dictionaries, and heuristic templates (12–14). These traditional systems operated through deterministic pipelines that combined lexical patterns and expert-defined rules to identify and remove personally identifiable information from clinical narratives. While effective in controlled or domain-specific contexts, they required extensive manual tuning and frequent updates to maintain accuracy, as they lacked the contextual understanding necessary to generalize across different hospitals, medical specialties, and writing styles (6, 15). This motivated the transition toward data-driven NLP approaches capable of leveraging linguistic context and statistical regularities to perform anonymization with higher robustness and scalability.

Moreover, anonymizing unstructured medical data is a necessary step for enabling research, including the development of NLP models and datasets that can meet privacy and security standards in healthcare. Tasks such as clinical outcome prediction, disease diagnosis, and patient monitoring require large amounts of annotated data, yet privacy concerns often restrict access to such data. This limitation is pronounced for Brazilian Portuguese, where a lack of publicly available, high-quality anonymized medical records impedes progress in building and evaluating NLP resources for medical applications (7, 8, 16–18).

To address these challenges, we present AnonyMed-BR, a new dataset specifically designed for the task of anonymizing medical records in Brazilian Portuguese. AnonyMed-BR fills a critical gap by providing annotated examples of medical text, with entities requiring anonymization clearly labeled and classified. Annotations include classes of personal and sensitive information, such as names, dates, identifiers and locations, enabling accurate entity detection and masking. By providing a structured resource for training NLP models capable of anonymizing unstructured medical records efficiently and in compliance with privacy regulations, we also enable the development of datasets and models for a wide range of tasks and applications within the medical domain, thereby stimulating medical research in NLP in Brazilian Portuguese and contributing to the larger goal of equitable advancements in language technologies.

To effectively anonymize sensitive unstructured medical data in Brazilian Portuguese, we investigate two distinct approaches. The first approach utilizes an extractive strategy based on NER with BERT-based models (19). The second approach adopts a generative strategy that leverages the T5-based (20) and GPT-based (21) models. The development and testing of these two distinct approaches in the newly created dataset offer valuable insights into their effectiveness in anonymizing unstructured medical data. By comparing extractive and generative strategies, this work not only advances the anonymization of Brazilian Portuguese unstructured medical records but also contributes to the broader effort of creating NLP resources adapted to the specific needs of the healthcare sector.

This work presents several novel contributions to the field of medical data anonymization. The key contributions of this study are listed below in order of significance. The first item represents the primary contribution of the study, while the subsequent items highlight secondary or supporting contributions:

- **Development of a new dataset for medical record anonymization:** A curated dataset tailored for the anonymization of unstructured medical records in Brazilian Portuguese.
- **Synthetic data generation:** Implementation of a data augmentation strategy employing GPT-4o to generate synthetic unstructured medical records from real-world data, enhancing the diversity and robustness of the dataset, addressing the limited availability of raw clinical data due to privacy constraints, and enabling the generation of large volumes of data necessary for fine-tuning robust NLP models without compromising patient privacy.
- **Generative models for anonymization tasks:** Proposal and evaluation of a generative anonymization approach, utilizing models such as T5 and GPT-4, to rewrite medical records with embedded tags for sensitive entities.
- **Evaluation of synthetic data quality:** Assessment of the quality and utility of synthetic data by comparing the performance of models fine-tuned exclusively on original data versus those fine-tuned exclusively on synthetic data.
- **LLM-based evaluation metric:** Introduction of an evaluation framework, where Gemini 2.5 Pro serves as a judge to assess anonymization quality, measure information loss due to masking, and estimate the difficulty of reidentifying masked personal information.
- **Pretraining vs. task-specific adaptation:** Analysis of the trade-offs between using a model pre-trained on medical domain data and one previously fine-tuned specifically for NER tasks, providing insights into model selection for anonymization challenges.

These contributions advance the research in medical data anonymization, offering both theoretical insights and practical methodologies applicable to real-world scenarios.

###  Related work

#### Anonymization of structured and unstructured medical records

The anonymization of medical data has been extensively studied, particularly for structured data, and can be broadly categorized into rule-based approaches, which rely on predefined patterns and heuristics, and model-based approaches, which use statistical or machine learning models (22). However, the rise of unstructured medical records and advances in NLP have shifted focus to anonymizing free text (4, 23–28), especially under stringent privacy regulations such as GDPR and LGPD (29). NER-based approaches, where models are fine-tuned to detect Personally Identifiable Information (PII), have shown strong performance in English clinical texts (30–35). These methods enable precise masking of sensitive entities, often achieving high F1 scores and human interpretability. Systematic reviews (5) summarize advancements while highlighting persistent limitations, such as dependency on large, annotated datasets and lack of contextual understanding.

#### Multilingual and Brazilian Portuguese resources

NER performance in underrepresented languages remains constrained by limited high-quality datasets. French (36) and Italian (37) datasets have enabled comparable F1 scores to English corpora, but dataset scarcity and annotation costs hinder progress. For Brazilian Portuguese, SemClinBr (16) and BioBERTpt (18) provide domain-specific corpora and pretrained models, while BRATECA (17) offers extensive real-world clinical notes. Despite these efforts, no dataset explicitly targets medical text anonymization in Brazilian Portuguese.

#### Generative approaches and large language models

Generative models, including GPT and LLaMA (38), have been applied to anonymization tasks, effectively handling context-dependent and ambiguous entities (39–47). Frameworks like DeID-GPT (23) replace sensitive information with generic tags, whereas our approach further classifies entities providing a more structured anonymization. While LLMs improve flexibility by allowing zero or few-shot learning, API-based models raise privacy concerns due to external data processing. Smaller generative models (20, 48) mitigate these risks by allowing local deployment without compromising sensitive information.

#### Gaps and contributions

Despite recent progress, several important gaps remain: (1) the scarcity of annotated datasets for underrepresented languages such as Brazilian Portuguese, which limits the development and evaluation of anonymization systems and may hinder the progress of the medical NLP field in Portuguese, due to the need for anonymized data to generate datasets and fine-tune models for diverse medical tasks; (2) the privacy and deployment concerns associated with API-based LLMs, as sensitive medical data must often be transmitted to external servers; and (3) the lack of structured anonymization capabilities in previous generative approaches, which typically mask sensitive information without classifying entities by type, reducing interpretability and downstream usability. Furthermore, no studies have explored LLM-as-a-Judge evaluation framework to assess anonymization quality beyond standard quantitative metrics.

Our method addresses these gaps by: (1) developing the first dataset explicitly designed for medical text anonymization in Brazilian Portuguese, (2) improving generative anonymization using smaller models that simultaneously mask and classify entities while preserving privacy through local deployment, and (3) introducing a scalable LLM-as-a-Judge evaluation framework to complement traditional metrics and enable systematic assessment.

## Methods

###  Task

Medical Data Anonymization is a critical task in ensuring privacy and compliance with regulations such as GDPR, LGPD, and HIPAA. The process involves removing or anonymizing Personally Identifiable Information from medical records to protect patient confidentiality while preserving the utility of data for research and analysis.

It is important to distinguish anonymization from related concepts such as de-identification and pseudonymization. De-identification refers to the process of removing or masking identifiers to reduce the risk of directly recognizing individuals, but it does not guarantee that the data cannot be re-identified. Pseudonymization, in contrast, replaces identifiers with reversible codes, allowing data to be re-linked to individuals under controlled conditions. Anonymization, as applied in this work, ensures that sensitive information cannot be traced back to any individual and is irreversible, meaning the original identifiers cannot be restored. This irreversibility is crucial for full compliance with data protection regulations and ensures that the dataset can be safely used for research and secondary analyses without risking patient re-identification.

The task is challenging due to the sensitive nature of medical information and the need to maintain the integrity of the data for secondary use. Techniques such as NER, and advanced methods such as LLMs are used to identify and anonymize sensitive details, including names, addresses, and other personal information.

Effective anonymization balances the need for privacy with the retention of valuable insights in the data, ensuring that sensitive information cannot be traced back to individuals while allowing researchers and practitioners to continue leveraging the data for advancements in healthcare.

###  Dataset

The dataset used in this experiment consists of 2,962 medical records, each containing various types of personally identifiable information, such as names, ids, dates, and locations. These records were selected to reflect a range of real-world scenarios encountered in Brazilian healthcare, including different PII formatting and diverse medical contexts.

In the next sections, we explain the steps taken to prepare the dataset for the task of medical data anonymization. It includes the preprocessing methods used to organize the data and the annotation process for identifying and labeling PIIs, ensuring the quality and suitability of the dataset for training and evaluation.

#### Dataset preprocessing

The clinical records used in this study were extracted from Electronic Health Records of 387 patients hospitalized at a Brazilian tertiary care hospital. The institution serves as a regional reference center and features an average length of stay of approximately seven days. It comprises a wide range of specialized medical wards—including nephrology, gastroenterology, cardiology, pulmonology, pediatrics, endocrinology, oncology, rheumatology, dermatology, infectious diseases, internal medicine, emergency medicine, clinical neurology, immunology, geriatrics, and hematology—as well as surgical wards such as general surgery, oncologic surgery, orthopedic surgery, cardiovascular surgery, thoracic surgery, trauma surgery, neurosurgery, vascular surgery, reconstructive plastic surgery, urologic surgery, otolaryngologic surgery, and ophthalmologic surgery. Additionally, the hospital also maintains beds in the intensive care unit (ICU).

This diversity of specialties ensures that the data reflect a broad spectrum of real-world clinical contexts, with the goal of constructing a high-quality dataset to support the development of robust transformer models for medical data anonymization, intended to generalize effectively across a wide range of medical domains. The preprocessing followed a structured two-step process: extraction of relevant segments from real records and augmentation through synthetic data generation.

To create the initial dataset, a Python script was developed to extract relevant segments from the medical records of each patient. These segments were carefully selected to include sensitive entities, such as patient names, ages, professions, dates, addresses, cities, medical IDs, and names of doctors or accompanying individuals. To ensure the data provided sufficient context for training, only segments longer than 1,300 characters were included, avoiding short notes or annotations that could still contain sensitive entities but lacked contextual richness. For each patient, a maximum of three segments were randomly chosen after shuffling the extracted segments. This process resulted in 1,075 samples derived from the original dataset.

Although real medical records provide a foundational dataset, their variability was limited due to the small number of patients (387). To address this, data augmentation was performed to increase the diversity of entities within the dataset. First, a set of 50 original samples was selected, and their entities were manually modified to protect the privacy of the individuals. These modified samples were then used as input for GPT-4o to generate synthetic data. The model produced samples that preserved the structure of the original records while introducing a broader variety of entities, such as new patient and doctor names, addresses, ages, professions, hospitals, emails, and phone numbers.

The synthetic samples underwent manual inspection to ensure quality. Poorly generated samples, such as those containing out-of-context text or the prompt itself, were discarded. The remaining high-quality synthetic records were shuffled separately for each of the 50 modified samples. For each modified sample, up to 40 synthetic examples were distributed across the training and test splits. This process resulted in the inclusion of 1,887 additional samples in the dataset.

To mitigate the risk of potential train/test leakage arising from generating multiple synthetic records from a small number of samples, we conducted a manual inspection and verified that, although the synthetic records often shared structural or stylistic patterns with their corresponding modified sample, the actual content differed substantially. This reduced the likelihood of overly similar examples appearing across splits. As a future improvement, we plan to generate synthetic data for training and testing from completely separate sets of modified samples, further minimizing any residual similarity between the splits.

**Figure 1:**
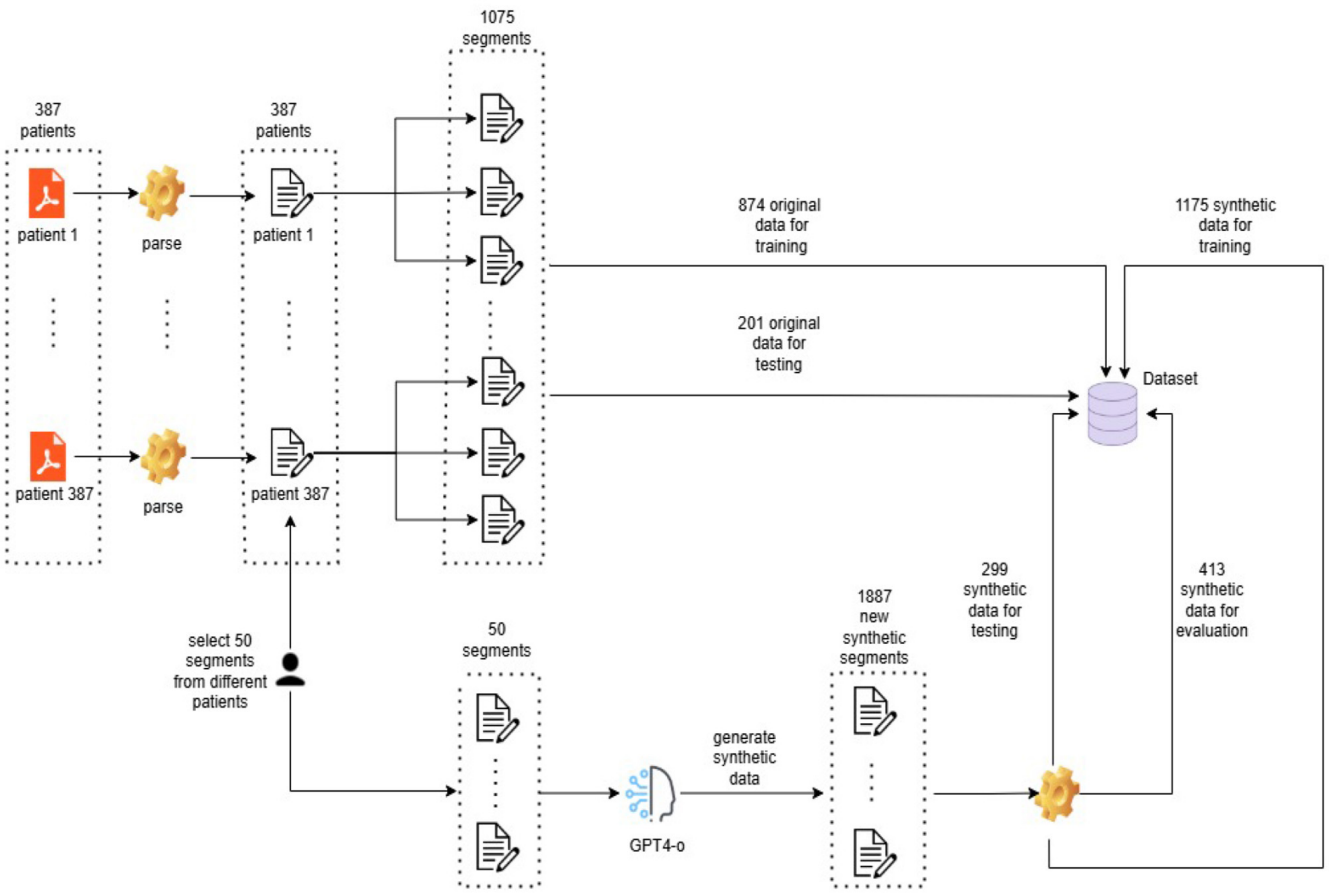
Diagram illustrating the dataset creation process, including the collection of real samples, generation of synthetic data, and final dataset composition for training, validation, and testing.

As illustrated in Figure 1, the final dataset combined 874 real training samples with 1,175 synthetic training samples. Additionally, 413 synthetic samples were reserved for evaluation. The test set consisted of 201 real samples and 299 synthetic samples, resulting in a diverse and contextually rich dataset for model training and evaluation.

#### Dataset annotation

To ensure effective anonymization of medical records, we conducted a manual annotation process in which a human annotator reviewed both original and synthetic records, identifying sensitive information that required protection. Each entity that needed anonymization was:

1. tagged using a predefined format corresponding to specific entity categories; and
2. in the case of original records, sensitive entities were manually replaced with contextually similar fictitious ones.

The annotation covered a wide range of personally identifiable information, including:

- **Personal data**: age  < AGE>, phone numbers  < PHONE>, and email addresses  < EMAIL>.
- **Unique identifiers**: identification numbers  < IDNUM>, medical record numbers  < MEDICAL_RECORD>, and health plan information  < HEALTH_PLAN>.
- **Geographical information**: addresses  < STREET>, cities  < CITY>, states  < STATE>, ZIP codes  < ZIP>, countries  < COUNTRY>, and other locations  < LOCATION_OTHER>.
- **Healthcare entities and professionals**: hospitals  < HOSPITAL>, organizations  < ORGANIZATION>, patients  < PATIENT>, doctors  < DOCTOR>, and professions  < PROFESSION>.
- **Additional sensitive information**: dates  < DATE>, marital status, level of education, and religion, grouped under  < OTHER>.

The annotator followed strict guidelines to ensure the consistency and accuracy in identifying and labeling sensitive information. In ambiguous cases, predefined rules were applied to determine the most appropriate tag for an entity. Each entity was enclosed within specific tags using the format  < TAG> before the entity and  < /TAG/> after it. To maintain uniformity and facilitate task evaluation, single-word entities were annotated individually, while multi-word entities, such as city names and hospital names, were split into separate tags. For example, a location such as “São Paulo” was annotated as  < CITY>São < /CITY/> < CITY>Paulo < /CITY/>, and a hospital such as “Hospital Santa Clara” was marked as  < HOSPITAL>Hospital < /HOSPITAL/> < HOSPITAL>Santa < /HOSPITAL/> < HOSPITAL>Clara < /HOSPITAL/>.

However, an exception is made for entities in the city-state format, where multiple tags are necessary to accurately distinguish different entity types within a single expression, even when no whitespace separates them. For example, when annotating a location such as “ITU-SP,” it is necessary to distinguish between the city and the state. Instead of applying a single tag to the entire term, the annotation follows the structure  < CITY>ITU < /CITY/>- < STATE>SP < /STATE/>, ensuring that each entity is correctly identified.

During annotation, except for the replaced sensitive entities in the original records, the content of the medical record was preserved without any modification or addition of extra text. Special care was taken with synthetic records generated by language models, as they occasionally contained snippets of text related to the prompt used for generation. Any such irrelevant content was removed to ensure data integrity. These guidelines ensured that all annotations were standardized, reducing inconsistencies and improving the quality of the labeled dataset.

To assess the reliability of the annotation process, a subset of the data was independently reviewed by a second annotator. The inter-annotator agreement was measured using Cohen's Kappa coefficient, calculated with the scikit-learn library. The result showed a **95% agreement**, indicating a high level of consistency in the annotation process.

This manual annotation process was essential for constructing a well-structured dataset, providing a solid foundation for the development of efficient transformer-based models for medical data anonymization in Brazilian Portuguese. The replacement of sensitive entities in the original data for similar fictitious ones was carried out to enable the safe sharing of the dataset with the scientific community, while ensuring the protection of the privacy of all individuals and institutions involved.

#### Dataset statistics

To evaluate the performance of models on anonymization of clinical text, we constructed a dataset composed of both real and synthetic medical records, totaling 2,962 samples. These were distributed across training (2,049 samples), validation (413 samples), and test (500 samples) sets. Each subset may include a mix of real and synthetic data (Table 1), with the training set containing the highest proportion of real records (874 real vs. 1,175 synthetic), while the validation set comprises only synthetic records. The test set combines 201 real and 299 synthetic samples to enable a fair and diverse model evaluation.

**Table 1:** Dataset split into training, validation (Dev), and test subsets, showing the number of real and synthetic samples in each set.

**Split**	**Real**	**Synthetic**	**Total**
Train	874	1,175	2,049
Validation	0	413	413
Test	201	299	500

Table 2 summarizes the textual and structural properties of the dataset. Real records tend to be significantly longer, with an average word count of 650.35 in the training set and 778.83 in the test set, compared to synthetic samples with average lengths of 254.23 and 264.04 words, respectively. This discrepancy reflects the natural verbosity of clinical narratives compared to their automatically generated counterparts.

**Table 2:** Dataset statistics for train, validation, and test sets.

**Dataset**	**Metric**	**Real**	**Synthetic**	**Total**
Train	Max words	4,022	637	–
	Min words	120	56	–
	Avg of words	650.35	254.23	423.20
	Avg of entities	28.15	22.37	24.84
	Avg entity density	5%	10%	8%
Validation	Max words	–	684	–
	Min words	–	678.6	–
	Avg of words	–	248.06	248.06
	Avg of entities	–	22.01	22.01
	Avg entity density	–	11%	11%
Test	Max words	3,685	566	–
	Min words	226	71	–
	Avg of words	778.83	264.04	470.99
	Avg of entities	30.95	22.67	26.0
	Avg entity density	4%	10%	8%

The table includes the maximum, minimum, and average word count per record, the average number of entities per record, and the average entity density, calculated as the ratio of the number of entities to the total word count in a record.

In terms of content richness, the number of named entities per record is also higher in real data (28.15 in train and 30.95 in test) than in synthetic samples (around 22 on average). However, synthetic records exhibit a higher entity density—a metric defined as the ratio of named entities to total word count in a record. For example, the training set shows an average entity density of 10% for synthetic data versus 5% for real data. This suggests that synthetic examples are more compact but more densely packed with identifiable entities, potentially increasing the difficulty of the task.

Entity-type distribution across dataset splits is presented in Table 3. The most frequent entity types across all splits include DATE, DOCTOR, and PATIENT, consistent with typical patterns in clinical documents. Nonetheless, differences are observed between real and synthetic subsets. For instance, real data emphasizes certain categories like DATE and DOCTOR more prominently, while synthetic samples include a broader set of PII types, such as EMAIL, STREET, and HEALTH_PLAN, which are absent or rare in real data.

**Table 3:** Percentage of entity frequency across dataset splits (train, validation, and test), separated into real and synthetic samples.

**Dataset**	**Split**	**Date**	**Doctor**	**Patient**	**Phone**	**City**	**Idnum**	**Age**	**Profession**	**Hospital**	**Medical_record**	**Street**	**Email**	**Location_other**	**Other**	**Organization**	**State**	**Country**	**Health_plan**	**Zip**
Train	Total	27.69%	21.25%	13.94%	4.90%	4.99%	4.80%	4.49%	3.03%	4.72%	1.97%	2.48%	1.82%	1.20%	1.30%	0.39%	0.92%	0.07%	0.02%	0.01%
	Real	46.35%	22.24%	11.37%	0.47%	3.35%	3.12%	3.73%	0.97%	2.94%	2.12%	–	–	0.28%	1.41%	0.61%	0.93%	0.11%	0.01%	–
	Synthetic	10.24%	20.33%	16.35%	9.05%	6.52%	6.38%	5.20%	4.96%	6.38%	1.84%	4.81%	3.53%	2.06%	1.20%	0.18%	0.91%	0.03%	0.02%	0.02%
Validation	Total	9.15%	22.25%	16.55%	9.28%	6.20%	6.46%	5.10%	4.97%	6.07%	1.64%	4.19%	3.64%	2.45%	0.38%	0.47%	1.08%	0.04%	–	0.04%
	Real	–	–	–	–	–	–	–	–	–	–	–	–	–	–	–	–	–	–	–
	Synthetic	9.15%	22.25%	16.55%	9.28%	6.20%	6.46%	5.10%	4.97%	6.07%	1.64%	4.19%	3.64%	2.45%	0.38%	0.47%	1.08%	0.04%	–	0.04%
Test	Total	29.54%	19.94%	13.44%	4.62%	4.89%	4.69%	4.15%	3.22%	4.89%	2.02%	2.74%	1.71%	1.19%	1.48%	0.73%	0.75%	–	–	0.01%
	Real	50.27%	19.97%	11.24%	0.16%	2.81%	2.93%	3.22%	1.40%	2.19%	2.17%	–	–	0.16%	1.67%	1.06%	0.76%	–	–	–
	Synthetic	10.50%	19.92%	15.46%	8.72%	6.79%	6.31%	5.00%	4.90%	7.38%	1.87%	5.25%	3.28%	2.14%	1.30%	0.43%	0.74%	–	–	0.01%

These natural variations in entity frequency and density across splits and sources are valuable for developing robust models. By exposing anonymization systems to diverse entity contexts and distributions, both in terms of frequency and structural placement, the dataset promotes better generalization and performance across heterogeneous real-world scenarios.

###  Models

In the context of anonymizing medical records using NLP models, selecting the appropriate model architecture is crucial for ensuring both performance and scalability. In this work, we employed three different types of transformer-based models tailored to our specific task: encoder-only (BERT-based models), encoder-decoder (T5-based models), and decoder-only (GPT-based models). This allows us to explore the strengths of each architecture in handling the complexities of the task. The following subsections describe each model type.

#### BERT-based models

Bidirectional Encoder Representations from Transformers (BERT) (19) model is a widely used transformer-based language model pre-trained on large corpora using a masked language modeling objective, which allows it to capture deep bidirectional representations of text.

In this study, we utilize four BERT-based models: BERTimbau (49),^1^ pierreguillou/bert-large-cased-pt-lenerbr (BERTimbau-leNER),^2^ Multilingual BERT (mBERT) (50),^3^ and BioBERTpt (18).^4^ BERTimbau contains approximately 335 million parameters and is a Brazilian Portuguese variant of BERT, pre-trained on a large corpus of Portuguese texts, making it well-suited for a wide range of natural language processing tasks in Portuguese. The pierreguillou/bert-large-cased-pt-lenerbr model builds upon BERTimbau large and is further fine-tuned on the LeNER-Br dataset (51), which focuses on NER applied to the legal domain, demonstrating strong performance in identifying and categorizing entities within specialized contexts. Multilingual BERT, with approximately 110 million parameters, is a language-agnostic version of BERT pre-trained on text from 104 languages, enabling its use in multilingual tasks, including those in Brazilian Portuguese. Lastly, BioBERTpt is a domain-specific model derived from mBERT, further pre-trained on Brazilian electronic health records. This additional pre-training significantly enhances its performance in clinical NLP tasks, making it particularly effective for applications requiring domain-specific language understanding.

#### T5-based models

Text-to-Text Transfer Transformer (T5) (20) is a language model that frames all NLP tasks as text-to-text problems, enabling consistent use of the same structure for tasks such as translation, summarization, question answering, and classification. Pretrained on the Colossal Clean Crawled Corpus (C4), a large web-based dataset, T5 demonstrated state-of-the-art performance across multiple benchmarks and is available in five configurations (Base, Small, Large, 3B, and 11B).

In this study, we use the ptt5-v2-base model (52),^5^ an adaptation of the T5-base architecture (≈220M parameters) pre-trained on large Brazilian Portuguese text corpora. This model builds on the strengths of the original T5 model while addressing the linguistic nuances of Brazilian Portuguese.

#### GPT-based models

The Generative Pre-trained Transformer (GPT) models (21) are a series of autoregressive language models designed for generating human-like text. Built on the Transformer architecture (53), GPT models have significantly advanced natural language processing by achieving state-of-the-art results across a wide range of tasks with minimal fine-tuning. Their ability to perform well in zero-shot and few-shot learning scenarios has sparked significant interest, as they can handle new tasks with little to no task-specific training.

GPT models excel in several key applications, including text generation, question answering, text completion, and even code generation. They produce coherent and contextually relevant text, handle complex queries with high accuracy, and generate high-quality summaries and translations. Their success across various domains demonstrates their ability to generalize effectively, making GPT models a powerful tool for many real-world NLP applications.

In this study, we use two variants of the GPT-4 model: GPT-4o and GPT-4o mini. The GPT-4o is a multimodal model capable of processing and generating text, images, and audio, with an impressive context window of 128k tokens that offers enhanced performance for various applications. The GPT-4o mini, a more compact version, maintains high performance with reduced computational requirements, processing text and images at a lower cost, making it more accessible especially for large-scale text processing scenarios.

We note that companies developing closed-source LLMs do not disclose the exact parameter counts of their models. While the general understanding in the community is that these architectures contain hundreds of billions to potentially trillions of parameters, no precise figures are publicly available.

###  Experiments

In this section, we present two distinct approaches for anonymizing sensitive information in medical records: an extractive strategy based on NER and a generative strategy. Both methods are designed to ensure privacy while preserving the usability and coherence of the anonymized data, as well as providing an efficient framework for evaluating model performance.

The experiments with BERT and T5 models were conducted on a Tesla T4 with 16 GB of memory. For fine-tuning, we used a learning rate of 5e-5, a batch size of 4, and full precision (fp32). The models were trained for 3 epochs. For GPT models, evaluation was carried out using few-shot learning with three training set examples included in the prompt. Generation parameters were set to a top-p of 1 and a temperature of 0 to ensure deterministic outputs. The system prompt is shown in Appendix Figure 4, and the complete prompt including the three few-shot examples is available in the project's GitHub repository.

#### NER-based anonymization

In the first approach, we employ an extractive anonymization strategy using BERT-based models to perform NER on medical records. The primary goal was to identify entities containing personal or private information, such as names, dates, or locations, and mask them to ensure privacy. The process involves providing the medical record text as input to the model, which returns a list of tuples for each identified entity, including the extracted word, its corresponding class, and its position in the text. Using this information, the original text is modified by replacing the identified words with their respective class tags, effectively anonymizing sensitive information while preserving the structure of the document.

#### Generative-based anonymization

The second approach uses T5-based and GPT-based models to identify sensitive information in medical records. The motivation for exploring this generative strategy is that most recent and more powerful NLP models are generative in nature, and we aimed to investigate their capabilities for the anonymization task. Inspired by existing approaches in the literature, we extend them by not only identifying sensitive entities but also classifying each entity into a specific category. In this approach, the model rewrites the original text while embedding specific tags around words containing private information. By adding < TAG> and < /TAG/> markers to these entities, the models highlight the sensitive data while keeping the rest of the text unchanged, maintaining its original meaning and context. The tagged output serves two main purposes: it allows for easy identification and evaluation of the detected entities, and it provides a straightforward method to mask private information by simply replacing the word between tags for the tag itself, ensuring the data complies with privacy regulations.

###  Metrics

To ensure a comprehensive evaluation of the anonymization methods, we employ both traditional quantitative metrics and qualitative assessments using LLMs. The combination of these approaches allows us to measure not only the accuracy of anonymization but also its impact on text utility and privacy preservation. In this section, we describe the key metrics used in our evaluation, including precision, recall, and F1 score, as well as a novel evaluation methodology leveraging LLMs to provide deeper insights into the quality of anonymized medical records.

#### F1 score

The performance of the anonymization methods are evaluated using precision, recall, and F1 scores as the key metrics, ensuring comprehensive removal and classification of PII while maintaining the semantic structure of the text. In this context, precision refers to the proportion of correctly classified and anonymized PII out of all the entities that were anonymized by the model, indicating how accurate the model's anonymization is. Recall, on the other hand, measures the proportion of actual PII that the model successfully classified and anonymized, reflecting its ability to detect all sensitive information. The F1 score provides a harmonic mean of precision and recall, offering a balanced metric for assessing the model's overall performance.


$$\begin{array}{c} \text{Precision}=\frac{\text{True Positive}}{\text{True Positive}+\text{False Positive}} \end{array}$$ (1)

$$\begin{array}{c} \text{Recall}=\frac{\text{True Positive}}{\text{True Positive}+\text{False Negative}} \end{array}$$ (2)

$$\begin{array}{c} \text{F1 Score}=\frac{2\times \text{Precision}\times \text{Recall}}{\text{Precision}+\text{Recall}} \end{array}$$ (3)

#### LLM-as-a-Judge

The LLM-as-a-Judge technique leverages the advanced capabilities of LLMs to evaluate the quality of outputs generated by other language models. Applying their sophisticated language understanding, these models can effectively evaluate unstructured data, providing valuable insights and scores for tasks that are challenging to measure with traditional methods. This approach simplifies the performance evaluation of language models, playing a crucial role in the development and optimization of AI systems.

In this project, we apply the LLM-as-a-Judge methodology to evaluate the performance of medical record anonymization systems relying on the criteria proposed by Mozes and Kleinberg (54). Using the Gemini 2.5 Pro model as the judge, we analyze three versions of the same medical record:

- The original version, without any anonymization.
- The version anonymized manually by a human expert.
- The version anonymized automatically by the language model.

For each version, Gemini 2.5 Pro assigns a score, ranging from 0 to 10, and generates a brief paragraph justifying the rationale behind each score for the three criteria proposed as follows:

- **Technical performance:** Measures the effectiveness of the anonymization in identifying and masking the correct sensitive entities within the text.
- **Information loss:** Evaluate whether anonymization compromises text coherence and quality, potentially affecting its use for training NLP models in clinical applications.
- **Re-identification risk:** Estimates the likelihood that anonymized information could still be reverse-engineered or linked back to identifiable individuals, indicating the robustness of the anonymization against potential re-identification attempts.

After completing the individual evaluations, the model compiles the generated paragraphs into a detailed report for each analyzed criteria, providing relevant insights into key strengths, weaknesses, and common errors observed during the anonymization process. This approach allows for a precise comparison between human and automatic anonymization, focusing on privacy preservation and maintaining the utility of medical data.

## Results

The performance of the different anonymization approaches evaluated on a set of medical records is summarized in Table 4.

**Table 4:** Performance metrics of different anonymization approaches evaluated on a test dataset of 500 medical records.

**Model**	**Split**	**F1 Score**	**Recall**	**Precision**
BERTimbau-leNER	Overall	**0.9270**	**0.9351**	**0.9260**
	Real	0.9007	0.9033	0.9097
	Synthetic	**0.9447**	**0.9564**	**0.9369**
GPT-4o	Overall	0.9195	0.9233	0.9194
	Real	**0.9356**	**0.9375**	**0.9391**
	Synthetic	0.9089	0.9140	0.9064
ptt5-v2	Overall	0.8748	0.8645	0.8918
	Real	0.8794	0.8705	0.8955
	Synthetic	0.8718	0.8605	0.8893
GPT-4o mini	Overall	0.8595	0.8887	0.8480
	Real	0.8264	0.8578	0.8337
	Synthetic	0.8759	0.9040	0.8550
BioBERT	Overall	0.8523	0.8605	0.8512
	Real	0.8505	0.8503	0.8626
	Synthetic	0.8535	0.8674	0.8435

The table shows Recall, Precision, and AVG F1 Score for each approach. Bold values denote the best-performing results for each evaluation setting.

The BERTimbau-leNER model achieved the best overall performance among the evaluated models, reaching an average F1 score of 0.9270, with a precision of 0.9260 and a recall of 0.9351 on the test dataset. A segmented analysis revealed a notable performance difference between original and synthetic records. On original records, the model achieved an F1 score of 0.9007, while on synthetic records, this value was significantly higher, reaching 0.9447. This suggests that the model benefits from regular linguistic patterns and less noise presented in the synthetic data. The entity-level analysis in Table 5 shows consistently high performance for well-defined and frequent entities such as AGE, CITY, DATE, DOCTOR, and PATIENT, all scoring around 0.9000. Conversely, more challenging entities like ORGANIZATION, LOCATION_OTHER, and PROFESSION exhibited lower F1 scores, particularly in original records—with a notable underperformance for LOCATION_OTHER, which reached only 0.1333, suggesting the model struggles to capture more open or less frequent contexts. Interestingly, these entities showed significant improvement on synthetic data, especially PROFESSION, which jumped from an F1 of 0.6101 on original records to 0.9397 on synthetic ones. These results indicate that while BERTimbau-leNER is highly effective at anonymizing most entities, its performance is sensitive to the linguistic variability present in original data, particularly for lower-frequency or more ambiguous entities.

**Table 5:** Performance per entity type for all evaluated models, showing how each approach handles different entities within the anonymization task.

**Model**	**Split**	**Date**	**Doctor**	**Patient**	**Phone**	**City**	**Idnum**	**Age**	**Profession**	**Hospital**	**Medical_record**	**Street**	**Email**	**Location_other**	**Other**	**Organization**	**State**	**Country**	**Health_plan**	**Zip**
BERTimbau-leNER	Real	0.8957	0.9120	**0.9724**	**1.0000**	0.9302	0.9125	**0.9863**	0.6101	**0.7599**	0.9926	–	–	0.1333	**0.6384**	0.6253	0.9907	–	–	–
	Synthetic	0.9562	**0.9744**	0.9853	0.9816	0.9525	0.9408	0.9918	**0.9397**	**0.9320**	0.7770	**0.9206**	0.8526	0.8402	0.6167	0.2063	0.9773	–	–	0.0000
GPT-4o	Real	**0.9398**	**0.9960**	0.9731	0.7778	**0.9437**	**1.0000**	0.9831	**0.6141**	0.2258	**1.0000**	–	–	0.4333	0.2757	**0.8802**	**1.0000**	–	–	–
	Synthetic	0.9587	0.9708	0.9806	0.8246	**0.9703**	**0.9598**	**0.9927**	0.9008	0.4766	0.8415	0.8726	**0.9985**	0.6584	**0.6264**	**0.9016**	**1.0000**	–	–	**1.0000**
ptt5-v2	Real	0.9053	0.9100	0.9270	**1.0000**	0.8629	0.9330	0.9740	0.1563	0.4863	0.9185	–	–	0.1333	0.0000	0.0500	0.6918	–	–	–
	Synthetic	0.9436	0.9131	0.9382	0.7501	0.8516	0.8769	0.9522	0.8369	0.8446	0.7295	0.7783	0.8888	0.3787	0.0000	0.0000	0.3500	–	–	0.0000
GPT-4o mini	Real	0.8727	0.9524	0.9134	**1.0000**	0.8237	0.9897	0.9128	0.3875	0.2136	0.9255	–	–	**0.5556**	0.4005	0.6810	0.8421	–	–	–
	Synthetic	0.9599	0.9351	0.9354	0.9242	0.9527	0.9584	0.9868	0.8489	0.5516	**0.8552**	0.8375	**0.9985**	0.5880	0.4693	0.5556	0.9773	–	–	**1.0000**
BioBERT	Real	0.8961	0.8803	0.9654	**1.0000**	0.9301	0.1173	0.9824	0.2688	0.4502	0.8914	–	–	0.1667	0.5442	0.1000	0.9352	–	–	–
	Synthetic	**0.9657**	0.9208	**0.9817**	**0.9640**	0.9530	0.2104	0.9896	0.9387	0.6843	0.6270	0.9198	0.2431	**0.8556**	0.5992	0.0952	0.9545	–	–	0.0000

Bold values denote the best-performing results for each evaluation setting.

Regarding the performance of the GPT-4o model, it achieved an overall F1 score of 0.9195, with a recall of 0.9233 and a precision of 0.9194, demonstrating strong capability in accurately identifying sensitive entities within medical records. When analyzing performance across data types, GPT-4o performed better on real data (F1 = 0.9356, Precision = 0.9391, Recall = 0.9375) compared to synthetic data (F1 = 0.9089, Precision = 0.9064, Recall = 0.9140), achieving the highest score among all evaluated models. This suggests that while the model generalizes well to both, it is more accurate when dealing with real-world data, possibly due to its significantly larger scale, broader pretraining and stronger generalization capabilities. These factors likely enable GPT-4o to better handle the variability, ambiguity, and inconsistencies commonly found in genuine medical records. At the entity level, GPT-4o exhibited consistently high performance for most categories, even achieving perfect scores (F1 = 1.0000) for entities such as IDNUM, MEDICAL_RECORD, STATE, and ZIP. However, similar to BERTimbau-leNER, performance was comparatively lower for more ambiguous categories such as HOSPITAL and LOCATION_OTHER, highlighting persistent challenges in disambiguating certain entity types.

The ptt5-v2 model demonstrated moderate performance in the anonymization task, achieving an overall F1 score of 0.8748, with a precision of 0.8918 and a recall of 0.8645. Its recall indicates a reasonable ability to identify sensitive entities, although the model tends to be slightly more conservative, favoring precision over recall. When evaluating the model separately on real and synthetic data, the performance remained relatively stable, with an F1 score of 0.8794 on real data and 0.8718 on synthetic data. Entity-level analysis reveals strong results for common entities such as AGE (F1 = 0.9740) and PHONE (F1 = 1.0000), but significant weaknesses on less frequent entities like ORGANIZATION and OTHER, where the model failed to make correct predictions. Additionally, entities such as LOCATION_OTHER, PROFESSION, and STATE also exhibited lower F1 scores, indicating that the model struggles with rarer or more ambiguous terms. Despite these limitations, ptt5-v2 presents a consistent and reliable performance for the most frequent and structurally well-defined entities within the dataset.

The GPT-4o mini model also demonstrated moderate performance in the anonymization task, achieving an overall F1 score of 0.8595, with a precision of 0.8480 and recall of 0.8887. When analyzing the results separately on original and synthetic medical records, the model showed higher effectiveness on synthetic data, obtaining an F1 score of 0.8759 compared to 0.8264 on original records, indicating better generalization in the synthetic domain. The precision and recall on synthetic samples were 0.8550 and 0.9040, respectively, while on original data these metrics were 0.8337 and 0.8578. Detailed evaluation by entity type revealed that the model performed exceptionally well on entities such as PHONE (F1 = 1.0000 on original data), DOCTOR, and IDNUM, achieving F1 scores above 0.9 in both data types. However, performance was notably lower for some categories such as HOSPITAL and PROFESSION, with F1 scores below 0.6000 in original samples, suggesting potential challenges in these specific entity types. In contrast to GPT-4o and similar to other smaller models, GPT-4o mini performed better on synthetic data, suggesting that model size—and consequently the number of parameters—may be a key factor in achieving the level of generalization needed to handle real-world medical records.

The BioBERT model ranked last overall, with performance only slightly below that of GPT-4o mini, with an F1 score of 0.8523, a recall of 0.8605, and a precision of 0.8512. When analyzing performance across original and synthetic samples, the model showed slightly better results on synthetic data (F1 = 0.8535) compared to original records (F1 = 0.8505), suggesting a relatively consistent behavior across test data. Despite strong performance in common entities such as AGE, PATIENT, DATE, and PHONE, where most F1 scores exceeded 0.9000, the model struggled significantly with critical identifiers like IDNUM (F1 = 0.1173 on original and 0.2104 on synthetic) and ORGANIZATION (F1 = 0.1000 on original and 0.0952 on synthetic). Additionally, ZIP codes were entirely missed (F1 = 0.0000). Entities like HOSPITAL and OTHER also showed moderate to poor results, particularly in original data. These findings suggest that while BioBERT is capable of capturing frequent and well-represented entities, it presents some limitations in its applicability to anonymization tasks in Brazilian Portuguese medical records.

When comparing the anonymization approaches evaluated in this study, BERTimbau-leNER and GPT-4o clearly stand out as the top-performing models across most metrics. BERTimbau-leNER achieved the highest overall score, driven primarily by its performance on synthetic data. On the other hand, GPT-4o demonstrated the best performance on real data, suggesting a remarkable ability of the model to handle the variability and complexity inherent to real-world medical records, surpassing BERTimbau-leNER in this context. Despite having slightly lower results on synthetic data, GPT-4o maintains strong overall performance, highlighting its robustness across different data formats.

The ptt5-v2 model performed moderately, with balanced metrics on both real and synthetic datasets, demonstrating consistency but not reaching the top scores achieved by GPT-4o and BERTimbau-leNER. Similarly, GPT-4o mini also showed moderate performance, performing better on synthetic data than on real records. As previously discussed, BioBERT ranked lowest among the evaluated models, though only by a small margin, with minimal differences between its performance on real and synthetic data.

It is worth noting that some entity types are extremely rare in the test set. This natural imbalance in entity frequency can lead to extreme F1 scores (0.0 or 1.0) for these categories due to the very small number of instances. While these per-entity F1 values should be interpreted with caution, we chose to report all entity types to ensure transparency and completeness in the evaluation results.

###  Domain pretraining vs. task fine-tuning

This experiment aims to investigate the trade-offs between two strategies for improving performance in the context of medical data anonymization: (i) leveraging models pretrained on general language corpora but fine-tuned specifically for NER tasks, and (ii) using models pretrained on domain-specific (medical) data without explicit fine-tuning for NER. To that end, we compare BERTimbau, a general Brazilian Portuguese language model, against its NER fine-tuned variant, BERTimbau-leNER. In parallel, we compare mBERT, a multilingual model without domain-specific adaptation, against BioBERTpt, which is the same model pre-trained on Brazilian Portuguese biomedical texts.

The results in Table 6 clearly highlight the superior impact of task-specific fine-tuning compared to domain-specific pre-training alone. BERTimbau-leNER achieves a substantial improvement over BERTimbau, with an increase of 4.35% in F1 score (from 0.8883 to 0.9270), suggesting that fine-tuning on NER tasks is highly effective for the anonymization challenge, even without exposure to domain-specific biomedical texts. In contrast, a performance decrease is observed from domain pre-training. BioBERT achieves an F1 score of 0.8523 compared to 0.8759 for mBERT, which surprisingly results in a decrease of approximately 2.70% in F1 score. This counterintuitive result suggests that while domain-specific pre-training may provide richer representations for medical language, it is insufficient on its own to optimize performance on the task of medical data anonymization.

**Table 6:** Comparison between task-specific NER fine-tuning and domain-specific pre-training for medical data anonymization.

**Approach**	**Model**	**F1 score**	**Recall**	**Precision**
(i)	BERTimbau	0.8883	0.8962	0.8862
	BERTimbau-leNER	**0.9270**	**0.9351**	**0.9260**
(ii)	mBERT	0.8759	0.8823	0.8756
	BioBERT	0.8523	0.8605	0.8512

Bold values denote the best-performing results for each evaluation setting.

The results suggest that task-specific fine-tuning yields greater performance gains than domain-specific pre-training when used in isolation. However, the combination of both approaches, domain-adapted models further fine-tuned on NER tasks, may present a promising direction, potentially offering the best of both worlds.

###  Original vs. synthetic data

This experiment aimed to evaluate the quality and utility of synthetic medical records by comparing the performance of an mBERT model fine-tuned exclusively on real data to the same model fine-tuned exclusively on synthetic data for approximately 4,000 steps. The experiment was conducted on two distinct test scenarios: one containing only real medical records and another composed of a mix of real and synthetic records.

As shown in Table 7, surprisingly, the model fine-tuned exclusively on synthetic data outperformed the one fine-tuned on real data across both test scenarios. When evaluated solely on real medical records, the model fine-tuned on synthetic data achieved an F1 score of 0.8919, surpassing the model fine-tuned on real data that achieved an F1 of 0.880. This difference became even more pronounced when evaluated on the full test set (real + synthetic), where the model fine-tuned exclusively on synthetic data achieved an F1 score of 0.9257, compared to 0.8367 for the model fine-tuned on real data, a difference of almost 9 F1 points.

**Table 7:** Performance comparison between mBERT fine-tuned exclusively on real medical records and fine-tuned on synthetic medical records.

**Model**	**F1 score**	**Recall**	**Precision**	**Split**
mBERT-real	0.8804	0.8870	0.8858	Real
mBERT-syn	**0.8919**	**0.8990**	**0.8909**	Real
mBERT-real	0.8367	0.8376	0.8439	Overall
mBERT-syn	**0.9257**	**0.9359**	**0.9191**	Overall

Bold values denote the best-performing results for each evaluation setting.

The results of this experiment revealed an unexpected, yet highly relevant finding. The mBERT model fine-tuned exclusively on synthetic data not only performed better than the same model fine-tuned on real data across all metrics, but the synthetic-trained mBERT achieved comparable performance to the best models analyzed in this study, including GPT-4o and BERTimbau-leNER, both of which were fine-tuned using a combination of real and synthetic data, and a superior performance than mBERT also fine-tuned on the combination of real and synthetic data. This suggests that, under certain conditions, synthetic data can be a viable substitute or even a superior alternative to real data for training anonymization models, especially in scenarios constrained by data privacy concerns or limited availability of annotated real-world medical records.

Nevertheless, this result is counterintuitive and raises important questions. As future work, we propose a deeper investigation into the underlying causes of this outcome, including whether this effect holds for other models beyond mBERT and under different data distributions or anonymization tasks.

###  LLM-as-a-Judge evaluation

Table 8 presents the results of the evaluation of medical record anonymization systems using the *LLM-as-a-Judge* methodology, in which the Gemini 2.5 Pro model served as the judge. The anonymization systems were evaluated according to three criteria: technical anonymization performance, information loss, and human re-identification risk, following the framework proposed by Mozes and Kleinberg (54).

**Table 8:** LLM-as-a-Judge scores (0–10) to evaluate medical record anonymization based on performance, information loss, and re-identification risk.

**Model**	**Technical performance**	**Information loss**	**Re-identification risk**
GPT-4o	**8.64**	**8.83**	**7.13**
BERTimbau-leNER	7.65	8.53	6.29
ptt5-v2	6.40	6.29	5.69

Bold values denote the best-performing results for each evaluation setting.

Each model was evaluated on a test set of 200 real medical records, previously anonymized by both human experts and language models. As shown in Appendix Figure 8, for each record, Gemini 2.5 Pro evaluates the original version, the expert-anonymized version, and the model-anonymized version, assigning a score from 0 to 10 for each of the three criteria, accompanied by a paragraph explaining the rationale behind each score. In all cases, higher scores indicate better performance, reflecting stronger anonymization, greater preservation of data utility, and enhanced protection against re-identification. The table reports the average score across all 200 records.

The results show that GPT-4o achieved the highest average scores across all evaluated criteria. In particular, it excelled in information loss, achieving a score of 8.83, which reflects its ability to anonymize sensitive information while maintaining the clinical coherence and utility of the original content. This indicates that GPT-4o effectively replaces or masks identifiable data without introducing inconsistencies or degrading the semantic quality of the text.

In terms of technical anonymization performance, which evaluates how effectively the model classifies and removes personally identifiable information, GPT-4o again led with a score of 8.64, suggesting that it was consistently accurate in detecting and replacing various entity types. BERTimbau-leNER followed with a score of 7.65, showing competitive capabilities in entity recognition and substitution. The ptt5-v2 model, while scoring lower, still managed to meet basic anonymization standards, though with occasional failures in removing more subtle identifiers.

Regarding re-identification risk, which measures the potential for a human to infer sensitive information post-anonymization, GPT-4o also ranked highest (7.13), indicating that it produced outputs with fewer indirect clues that could enable re-identification. BERTimbau-leNER scored lower (6.29), due to occasional preservation of contextual clues, such as partial dates or fragments that may still hint at personal information. ptt5-v2 obtained the lowest score in this category (5.69), suggesting that its anonymizations were more vulnerable to residual privacy risks.

As previously mentioned, in addition to scoring, Gemini 2.5 Pro generated textual evaluations for each of the three criteria for every medical record. To support a qualitative analysis, the first 25 evaluations for each criterion (totaling 75 per model) were selected and summarized into reports, as illustrated in Appendix Figure 12. Each report aims to identify general trends observed in the evaluations, highlight recurring strengths and weaknesses in the model performance, and pinpoint key issues commonly mentioned. These qualitative insights complement the quantitative scores by offering a deeper understanding of model behavior, such as difficulties in anonymizing specific entities or frequent over-removal of clinical content.

This combined approach of quantitative scoring and qualitative feedback can be a robust tool for evaluating automated anonymization systems, especially in sensitive domains such as medical records, providing both measurable benchmarks and a richer interpretation of model behavior and limitations.

## Discussion

Our study evaluated different approaches to medical data anonymization using NLP models, with a focus on medical records in Brazilian Portuguese. Our findings provide relevant insights into the performance, limitations, and applicability of extractive and generative anonymization methods and offer a foundation for the development of privacy-preserving NLP tools tailored to the healthcare context.

Our results, with the best-performing models achieving F1 scores above 90, are in line with trends observed in international studies on medical text anonymization, where transformer-based models have also demonstrated strong performance (23, 32, 46). However, it is important to note that these studies rely on different datasets, languages, and annotation schemes, which makes direct numerical comparison difficult. Nonetheless, the consistent high performance across approaches reinforces that both fine-tuned models and large generative models can effectively identify and anonymize sensitive information in clinical narratives.

Among the evaluated models, BERTimbau-leNER and GPT-4o emerged as the top performers. BERTimbau-leNER achieved the highest F1 score on synthetic data, suggesting a strong adaptation to well-structured and less noisy inputs. On the other hand, GPT-4o demonstrated superior performance on real medical records, highlighting its ability to generalize to noisy, complex, and variable inputs. The model's large-scale architecture, extensive pretraining, and versatile generative capabilities may provide a significant advantage in handling realistic clinical scenarios.

Statistical testing confirmed these tendencies. The difference between models was not statistically significant when considering all data combined (t = –1.63, *p* = 0.10), indicating comparable overall performance. However, when analyzed by data source, results revealed clear contrasts: on synthetic data, BERTimbau-leNER significantly outperformed GPT-4o (t = –5.63, *p* < 0.001), whereas on real medical records, GPT-4o achieved significantly higher scores (t = 2.74, *p* = 0.006). These findings reinforce that while fine-tuned transformer models such as BERTimbau-leNER are highly effective in controlled and structured domains, large generative models like GPT-4o exhibit stronger robustness and adaptability to real-world clinical text, where variability and noise are inherent.

Entity-level analysis further supports these findings: entities such as AGE, CITY, DATE, DOCTOR, and PATIENT showed consistently high detection rates, while entities like ORGANIZATION, LOCATION_OTHER, PROFESSION, and OTHER exhibited lower performance across models. These discrepancies point to ongoing challenges in the anonymization of ambiguous or infrequent entities and emphasize the need for further refinement, especially for entities that may pose higher re-identification risks.

A key contribution of this study lies in the comparison between domain-specific pre-training and task-oriented fine-tuning. Our results suggest that fine-tuning for NER tasks is more effective than domain pre-training when addressing anonymization challenges. This can be explained by the importance of supervised task alignment: the model learns to specialize in recognizing entities, regardless of domain, benefiting directly from NER-specific annotations. In contrast, pre-training on medical corpora improves its understanding of domain-specific vocabulary and context but does not provide the necessary task-specific knowledge required for optimal NER performance.

One particularly noteworthy and counterintuitive result was the high performance of the mBERT model fine-tuned solely on synthetic data, which achieved significantly better performance than its counterpart fine-tuned on real data, and even performed on par with the top models fine-tuned on the full dataset in this study. This outcome highlights the potential of synthetic data as a viable and, under certain conditions, superior resource for fine-tuning anonymization models. In scenarios where privacy regulations or data scarcity limit the availability of annotated real data, synthetic datasets may offer a practical and effective alternative.

To complement the evaluation, we introduced the LLM-as-a-Judge methodology, employing Gemini 2.5 Pro to perform a quantitative and qualitative evaluation of the anonymization process. This methodology enables a deeper understanding of model behavior, covering aspects such as information loss and re-identification risk of anonymized records. Notably, GPT-4o received the highest scores, aligning with its superior performance on real data in the traditional metrics.

The integration of large language models into the evaluation pipeline adds significant value by enabling qualitative assessment of the anonymization process, highlighting weaknesses and recurring errors, and also evaluating whether the anonymization makes sense in context and preserves the overall meaning of the text. This allows the system to detect cases where anonymized data could still reveal sensitive information due to surrounding context, or where excessive masking compromises the interpretability of the medical record.

Our findings also bring to light important considerations regarding model deployment. The observed trade-off between performance on real-world data and data privacy highlights the importance of selecting models according to the specific deployment context. For instance, although GPT-4o achieved the highest performance in handling the complexity of real clinical records, it relies on external APIs, which pose privacy concerns in healthcare environments. In contrast, BERTimbau-leNER, though not matching GPT-4o's performance on real data, still delivers solid results and offers a significant privacy advantage by supporting local deployment without the need for external data transmission.

Beyond performance and privacy, practical deployment in hospital environments is an important consideration. Models such as mBERT-syn and BERTimbau-leNER, in addition to their strong performance and privacy advantages, are relatively small and computationally efficient. They can be deployed locally with modest hardware requirements and can even run reasonably fast on machines without a GPU, which substantially reduces the cost and complexity of implementation. When coupled with a user-friendly interface and a workflow that converts clinical records into a format digestible by the NLP model, these systems could be deployed in hospitals to anonymize hundreds or even thousands of patient records daily, depending on the available hardware. This combination of efficiency, scalability, and privacy makes such models particularly suitable for real-world adoption in healthcare settings.

###  Limitations

While this work demonstrates promising results for anonymizing unstructured medical records in Brazilian Portuguese, several limitations should be noted. First, the results are specific to the medical domain in Brazilian Portuguese, and their generalization to other languages or textual domains remains untested. As future work, the evaluation of our models in new languages and diverse textual domains is recommended.

Second, anonymization is inherently imperfect: despite high F1 scores, there remains a residual risk that some sensitive information may not be fully masked, reflecting the trade-off between utility and privacy in practical applications. To mitigate this risk, periodic human audits of random samples can be performed to detect failures and improve the pipeline.

Third, although we adopt an LLM as judge (Gemini 2.5 Pro) to assess anonymization quality, our reliance on this paradigm should be viewed with caution (55–57). Recent studies highlight that LLM-as-a-Judge frameworks may suffer from issues such as self-preference bias, lack of domain-specific expertise, prompt-sensitivity, and limited generalizability, especially when the judge model is comparable in capability to the evaluated model (58, 59). Therefore, while LLM-based evaluation offers scalability, its outputs should be complemented by human oversight in deployment.

Finally, the synthetic data generation and augmentation strategies we employ, while beneficial for dataset diversity, may introduce unintended artifacts or biases that could affect downstream model behavior or evaluation (60). These should be monitored carefully as the technique is scaled.

## Conclusion

Our study contributes to the advancement of medical data anonymization in Brazilian Portuguese by presenting a comprehensive evaluation of anonymization strategies for medical records in Brazilian Portuguese, combining extractive and generative NLP approaches. By releasing a high-quality annotated dataset and an evaluation framework, we provide valuable tools to support further research on clinical data privacy.

Our findings highlight that while large models offer state-of-the-art performance on real data, locally deployable models strike a practical balance between anonymization quality and enhanced data privacy. These insights are particularly relevant for healthcare, where regulations restrict the external transmission of confidential health information.

Future work should focus on improving performance for underrepresented entities, advancing the use of synthetic data, and refining evaluation methods that integrate quantitative metrics, qualitative insights into anonymization effectiveness, and ethical dimensions of privacy protection.

## Data Availability

The datasets presented in this study can be found in online repositories. The names of the repository/repositories and accession number(s) can be found below: https://huggingface.co/datasets/Venturus/AnonyMED-BR.

## Footnotes

1
https://huggingface.co/neuralmind/bert-large-portuguese-cased

2
https://huggingface.co/pierreguillou/ner-bert-large-cased-pt-lenerbr

3
https://huggingface.co/google-bert/bert-base-multilingual-cased

4
https://huggingface.co/pucpr/biobertpt-clin

5
https://huggingface.co/unicamp-dl/ptt5-v2-base
