# Supplementary material for: Guardians of the data: NER and LLMs for effective medical record anonymization in Brazilian Portuguese
Source: Front Public Health. 2026 Jan 5;13:1717303. doi: 10.3389/fpubh.2025.1717303 (PMC12813187; doi:10.3389/fpubh.2025.1717303)
Supplement: Supplementary file 1 [file Data_Sheet_1.pdf]

# APPENDIX

## Anonymization Outputs

In this section, we present qualitative examples of medical records anonymized by our best-performing approaches. Figure 1 displays a comparison in Portuguese, while Figure 2 shows the corresponding English translations. In both cases, the original medical record is followed by the human-annotated version and outputs from GPT-4o, BERTimbau-leNER, and ptt5-v2. Correctly identified entities are highlighted in bold, and misclassified entities are shown in red.

```
- Original

 Mulher de meia-idade, 50 anos, professora, de nome Ana Carla Mota, telefone (21) 98476-2341 email ana.c.mota@exemplo.com,
 residente de Vila Isabel, Rio de Janeiro, apresenta queixa de aumento da frequência urinária,
 urgência miccional e disúria há 3 dias . Nega febre ou dor lombar . Refere episódios anteriores semelhantes .
 HD : Infecção do trato urinário recorrente . Coleta de urina realizada para exame de urina e urocultura .
 Iniciado tratamento empírico com antibiótico conforme protocolo de cistite não complicada .
 Elaborado e assinado por Dra . Letícia Barbosa de Souza, CRM 43820 em 08/8/2023 09:53

- Human Annotator

 Mulher de meia-idade, <AGE> anos, <PROFESSION>, de nome <PATIENT> <PATIENT> <PATIENT>, telefone <PHONE> <PHONE> email <EMAIL>,
 residente de <LOCATION_OTHER> <LOCATION_OTHER>, <CITY> <CITY> <CITY>, apresenta queixa de aumento da frequência urinária,
 urgência miccional e disúria há 3 dias . Nega febre ou dor lombar . Refere episódios anteriores semelhantes .
 HD : Infecção do trato urinário recorrente . Coleta de urina realizada para exame de urina e urocultura .
 Iniciado tratamento empírico com antibiótico conforme protocolo de cistite não complicada .
 Elaborado e assinado por Dra . <DOCTOR> <DOCTOR> <DOCTOR> <DOCTOR>, CRM <IDNUM> em <DATE> 09:53

- GPT-4o

 Mulher de meia-idade, <AGE> anos, <PROFESSION>, de nome <PATIENT> <PATIENT> <PATIENT>, telefone <PHONE> <PHONE> <PHONE> <PHONE> email <EMAIL>,
 residente de <LOCATION_OTHER> <LOCATION_OTHER>, <CITY> <CITY> <CITY>, apresenta queixa de aumento da frequência urinária,
 urgência miccional e disúria há 3 dias . Nega febre ou dor lombar . Refere episódios anteriores semelhantes .
 HD : Infecção do trato urinário recorrente . Coleta de urina realizada para exame de urina e urocultura .
 Iniciado tratamento empírico com antibiótico conforme protocolo de cistite não complicada .
 Elaborado e assinado por Dra . <DOCTOR> <DOCTOR> <DOCTOR> <DOCTOR>, CRM <IDNUM> em <DATE> 09:53

- BERTimbau-leNER

 Mulher de meia-idade , <AGE> anos , <PROFESSION> , de nome <PATIENT> <PATIENT> <PATIENT> , telefone (<PHONE>) <PHONE> email <EMAIL> ,
 residente de <LOCATION_OTHER> <LOCATION_OTHER> , <CITY> <CITY> <CITY> , apresenta queixa de aumento da frequência urinária ,
 urgência miccional e disúria há 3 dias . Nega febre ou dor lombar . Refere episódios anteriores semelhantes .
 HD : Infecção do trato urinário recorrente . Coleta de urina realizada para exame de urina e urocultura .
 Iniciado tratamento empírico com antibiótico conforme protocolo de cistite não complicada .
 Elaborado e assinado por Dra . <DOCTOR> <DOCTOR> <DOCTOR> <DOCTOR> , CRM <IDNUM> em <DATE> 09:53

- ptt5-v2

 Mulher de meia-idade, <AGE> anos, <PROFESSION>, de nome <PATIENT> <PATIENT> <PATIENT>, telefone <PHONE> <PHONE> email <EMAIL>,
 residente de <CITY> <CITY>, <CITY> <CITY> <CITY>, apresenta queixa de aumento da frequência urinária,
 urgência miccional e disúria há 3 dias . Nega febre ou dor lombar . Refere episódios anteriores semelhantes .
 HD : Infecção do trato urinário recorrente . Coleta de urina realizada para exame de urina e urocultura .
 Iniciado tratamento empírico com antibiótico conforme protocolo de cistite não complicada .
 Elaborado e assinado por Dra . <DOCTOR> <DOCTOR> <DOCTOR> <DOCTOR>, CRM <IDNUM> em <DATE> 09:53
```

**Figure 1.** Comparison of medical record anonymization in portuguese: the first shows the original medical record; the second, the version anonymized by a human specialist; followed by outputs from GPT-4o, BERTimbau-leNER, and ptt5-v2. Entities are highlighted in bold, and incorrectly classified entities are shown in red.

```
- Original

Middle-aged woman, 50 years old, teacher, named Ana Carla Mota, phone number (21) 98476-2341, email ana.c.mota@exemplo.com,
resident of Vila Isabel, Rio de Janeiro, complains of increased urinary frequency,
urinary urgency, and dysuria for the past 3 days. Denies fever or lower back pain. Reports similar previous episodes.
Diagnosis: Recurrent urinary tract infection. Urine collected for urinalysis and urine culture.
Empirical antibiotic treatment initiated according to the uncomplicated cystitis protocol.
Prepared and signed by Dr. Letícia Barbosa de Souza, CRM 43820 on 08/8/2023 at 09:53

- Human Annotator

Middle-aged woman, <AGE> years old, <PROFESSION>, named <PATIENT> <PATIENT> <PATIENT>, phone number <PHONE> <PHONE>, email <EMAIL>,
resident of <LOCATION_OTHER> <LOCATION_OTHER>, <CITY> <CITY> <CITY>, complains of increased urinary frequency,
urinary urgency, and dysuria for the past 3 days. Denies fever or lower back pain. Reports similar previous episodes.
Diagnosis: Recurrent urinary tract infection. Urine collected for urinalysis and urine culture.
Empirical antibiotic treatment initiated according to the uncomplicated cystitis protocol.
Prepared and signed by Dr. <DOCTOR> <DOCTOR> <DOCTOR> <DOCTOR>, CRM <IDNUM> on <DATE> at 09:53

- GPT-4o

Middle-aged woman, <AGE> years old, <PROFESSION>, named <PATIENT> <PATIENT> <PATIENT>, phone number <PHONE> <PHONE> <PHONE> <PHONE>, email <EMAIL>,
resident of <LOCATION_OTHER> <LOCATION_OTHER>, <CITY> <CITY> <CITY>, complains of increased urinary frequency,
urinary urgency, and dysuria for the past 3 days. Denies fever or lower back pain. Reports similar previous episodes.
Diagnosis: Recurrent urinary tract infection. Urine collected for urinalysis and urine culture.
Empirical antibiotic treatment initiated according to the uncomplicated cystitis protocol.
Prepared and signed by Dr. <DOCTOR> <DOCTOR> <DOCTOR> <DOCTOR>, CRM <IDNUM> on <DATE> at 09:53

- BERTimbau-leNER

Middle-aged woman, <AGE> years old, <PROFESSION>, named <PATIENT> <PATIENT> <PATIENT>, phone number (<PHONE>) <PHONE>, email <EMAIL>,
resident of <LOCATION_OTHER> <LOCATION_OTHER>, <CITY> <CITY> <CITY>, complains of increased urinary frequency,
urinary urgency, and dysuria for the past 3 days. Denies fever or lower back pain. Reports similar previous episodes.
Diagnosis: Recurrent urinary tract infection. Urine collected for urinalysis and urine culture.
Empirical antibiotic treatment initiated according to the uncomplicated cystitis protocol.
Prepared and signed by Dr. <DOCTOR> <DOCTOR> <DOCTOR> <DOCTOR>, CRM <IDNUM> on <DATE> at 09:53

- ptt5-v2

Middle-aged woman, <AGE> years old, <PROFESSION>, named <PATIENT> <PATIENT> <PATIENT>, phone number <PHONE> <PHONE>, email <EMAIL>,
resident of <CITY> <CITY>, <CITY> <CITY> <CITY>, complains of increased urinary frequency,
urinary urgency, and dysuria for the past 3 days. Denies fever or lower back pain. Reports similar previous episodes.
Diagnosis: Recurrent urinary tract infection. Urine collected for urinalysis and urine culture.
Empirical antibiotic treatment initiated according to the uncomplicated cystitis protocol.
Prepared and signed by Dr. <DOCTOR> <DOCTOR> <DOCTOR> <DOCTOR>, CRM <IDNUM> on <DATE> at 09:53
```

**Figure 2.** Comparison of medical record anonymization translated to english: the first shows the original medical record; the second, the version anonymized by a human specialist; followed by outputs from GPT-4o, BERTimbau-leNER, and ptt5-v2. Entities are highlighted in bold, and incorrectly classified entities are shown in red.

## GPT Prompt

In this section, we present the prompt used with the GPT-4o and GPT-4o mini models. Figure 3 illustrates the complete prompt structure in both Portuguese and English. This prompt was used together with three examples from the training dataset in a few-shot learning format to guide the models during the anonymization task. The complete version of the prompt is available in the source code provided in the project's GitHub repository.

```
Você é uma assistente que auxilia na identificação de entidades em textos médicos incluindo tags de marcação no texto original.
Você deve identificar somente as seguintes entidades, não crie novas entidades além das listadas aqui: "AGE", "PHONE", "EMAIL", "DATE",
"IDNUM", "MEDICAL_RECORD", "HEALTH_PLAN", "STREET", "CITY","ZIP", "STATE", "COUNTRY", "LOCATION_OTHER", "ORGANIZATION", "HOSPITAL",
"PATIENT", "DOCTOR", "USERNAME", "PROFESSION", "OTHER".
As tags possuem o seguinte formato: <PATIENT> </PATIENT/> <DOCTOR> </DOCTOR/> <DATE> </DATE/>.
Cada par de tags deve conter apenas uma palavra como mostram os exemplos abaixo.

You are an assistant designed to identify entities in medical texts by including markup tags in the original text.
You must identify only the following entities – do not create new ones beyond those listed here: "AGE", "PHONE", "EMAIL", "DATE",
"IDNUM", "MEDICAL_RECORD", "HEALTH_PLAN", "STREET", "CITY", "ZIP", "STATE", "COUNTRY", "LOCATION_OTHER", "ORGANIZATION", "HOSPITAL",
"PATIENT", "DOCTOR", "USERNAME", "PROFESSION", "OTHER".
The tags follow this format: <PATIENT> </PATIENT/>, <DOCTOR> </DOCTOR/>, <DATE> </DATE/>.
Each pair of tags must contain only one word, as shown in the examples above.
```

**Figure 3.** Prompt employed to guide the GPT-4o and GPT-4o-mini models during the evaluation of the *AnonyMed-BR* test set.

## LLM-as-a-Judge Prompt

In this section, we present the prompt used to guide the Gemini 2.5 Pro model for the LLM-as-a-Judge evaluation. Figure 4 shows the prompt in Portuguese, while Figure 5 displays the corresponding English translation. The prompt include the input format (Original, Human-Anonymized, Model-Anonymized), the three scoring criteria (Technical Performance, Information Loss, and Re-identification Risk), and the expected JSON output format.

```
Você é um especialista em anonimização de textos médicos e sua tarefa é avaliar a qualidade da anonimização de um prontuário médico baseado em três critérios que são:
Desempenho técnico da anonimização, Perda de Informação e Risco de reidentificação.

Entrada:
Você receberá três versões do mesmo prontuário médico:

Original: Prontuário médico sem anonimização.
Anonimizado por especialista: Versão do prontuário que foi anonimizada manualmente por um especialista humano.
Anonimizado por modelo: Versão do prontuário que foi anonimizada por um modelo de linguagem.

Tarefa:
Avalie a versão anonimizada pelo modelo de linguagem em relação ao prontuário original e à versão anonimizada pelo especialista, utilizando os seguintes critérios:

Desempenho técnico da anonimização: Avalie se todas as informações identificáveis (nomes, datas, endereços, telefone, nome de hospital, números de identificação de médicos e pacientes, etc.)
                                    foram corretamente removidas ou mascaradas. Score 0 (péssimo) a 10 (perfeito).

Perda de informação: Avalie se a anonimização preservou o máximo possível de informações úteis para análise clínica e treinamento de um modelo de deep learning em tarefas
                     médicas, mantendo a legibilidade e o valor semântico do texto comparado ao Original. Score 0 (informação completamente perdida) a 10 (mínima perda de informação).

Capacidade de reidentificação humana: Avalie se um humano poderia reidentificar o paciente com base nas informações restantes no prontuário anonimizado.
                                      Quanto maior o risco, menor a nota. Score 0 (altíssimo risco de reidentificação) a 10 (nenhuma chance de reidentificação).
Formato de saída:

Forneça a avaliação no seguinte formato JSON:
            {
              "Desempenho": {
                "score": <score>,
                "text": "<explicação detalhada da nota atribuída>"
              },
              "Perda": {
                "score": <score>,
                "text": "<explicação detalhada da nota atribuída>"
              },
              "Reidentificação": {
                "score": <score>,
                "text": "<explicação detalhada da nota atribuída e entidades problemáticas>"
              }
            }
Agora, analise os três prontuários fornecidos e gere a saída JSON. No campo de texto dê um diagnóstico preciso e aprofundado explicando em detalhes o porquê da nota recebida.
```

**Figure 4.** Prompt used to guide the Gemini 2.5 Pro model for the LLM-as-a-Judge evaluation. The prompt specifies the input format, the three scoring criteria, and the expected JSON output format.

```
You are a specialist in medical text anonymization, and your task is to evaluate the quality of anonymization of a medical record based on three criteria:
Technical anonymization performance, Information loss, and Risk of re-identification.

Input:
You will receive three versions of the same medical record:

Original: The un-anonymized medical record.
Anonymized by specialist: The version of the medical record manually anonymized by a human expert.
Anonymized by model: The version of the medical record anonymized by a language model.

Task:
Evaluate the model-anonymized version in relation to the Original and the specialist-anonymized version using the following criteria:

Technical anonymization performance: Assess whether all identifiable information (names, dates, addresses, phone numbers, hospital names, doctor and patient IDs, etc.) has been correctly
                                     removed or masked. Score 0 (very poor) to 10 (perfect).

Information loss: Assess whether the anonymization preserves as much clinically useful information as possible for analysis or for training deep learning models on medical tasks,
                  maintaining readability and semantic value compared to the Original. Score 0 (information completely lost) to 10 (minimal information loss).

Human re-identification risk: Assess whether a human could re-identify the patient based on the information remaining in the anonymized record. Higher risk corresponds to a lower score.
                              Score 0 (very high risk) to 10 (no chance of re-identification).
Output format:
Provide the evaluation in the following JSON format:

{
  "TechnicalPerformance": {
    "score": <score>,
    "text": "<detailed explanation of the assigned score>"
  },
  "InformationLoss": {
    "score": <score>,
    "text": "<detailed explanation of the assigned score>"
  },
  "ReidentificationRisk": {
    "score": <score>,
    "text": "<detailed explanation of the assigned score and any problematic entities>"
  }
}
Now, analyze the three provided medical records and generate the JSON output. In the text fields, provide a precise and in-depth diagnosis explaining in detail why the score was assigned.
```

**Figure 5.** English translation of the prompt used to guide the Gemini 2.5 Pro model for the LLM-as-a-Judge evaluation. The prompt specifies the input format, the three scoring criteria, and the expected JSON output format.

## LLM-as-a-Judge Outputs

In this section, we present sample evaluations generated by Gemini 2.5 Pro, based on the three criteria defined in the LLM-as-a-Judge framework: Technical Performance, Information Loss, and Re-identification Risk. Each assessment is conducted on a tuple consisting of the original medical record, a version anonymized by a human expert, and a version anonymized by a language model. Figure 6 illustrates these evaluations in Portuguese, while Figure 7 presents the corresponding English translations.


```
{'Performance': {
        'score': 10,
        'text': "O modelo de linguagem demonstrou um desempenho técnico perfeito na anonimização, replicando exatamente o resultado do especialista.
                 Todas as informações identificáveis presentes no prontuário original foram corretamente removidas ou mascaradas.
                 Especificamente, o nome da enfermeira ('Gabriela Cardoso') foi substituído pela tag genérica '<DOCTOR> <DOCTOR>',
                 o número do Coren ('329999') foi substituído por '<IDNUM>', e a data completa ('17/02/2017') foi substituída por '<DATE>'.
                 A hora ('15:46') foi mantida, o que é consistente com a anonimização do especialista e, na ausência da data completa,
                 representa um risco muito baixo de identificação. Não havia outros dados PII diretos no corpo do texto que precisassem de remoção,
                 e o modelo não introduziu falsos positivos. A única alteração mínima foram alguns espaços adicionais em torno de pontuação e tags,
                 como 'Enf . <DOCTOR> <DOCTOR> , Coren <IDNUM> em <DATE> 15:46', que não afetam a qualidade da anonimização em si."},


'Loss': {'score': 9,
         'text': "A anonimização realizada pelo modelo preservou quase que integralmente as informações úteis para análise clínica e
                  treinamento de modelos de deep learning. O corpo principal do prontuário, contendo as observações clínicas sobre o estado da paciente,
                  procedimentos ('broncoscopia'), exames físicos e escores (FUGULIN, Flebite, MORSE, BRADEN), permaneceu inalterado e legível,
                  mantendo seu valor semântico. A substituição das informações do profissional por tags genéricas é uma prática padrão que remove PII sem
                  comprometer o conteúdo clínico relevante para a paciente. A pequena perda de nota (de 10 para 9) deve-se a um erro de digitação/aglutinação
                  ('lesões depele' em vez de 'lesões de pele') introduzido pelo modelo e aos espaçamentos ligeiramente anormais na linha da assinatura
                  ('Enf . <DOCTOR> <DOCTOR> , Coren <IDNUM> em <DATE> 15:46'). Embora 'Enf.' tenha sido substituído por '<DOCTOR> <DOCTOR>',
                  o que é uma generalização, a categoria profissional 'Enf.' foi mantida, o que é positivo. Essas são questões menores que não afetam
                  substancialmente a utilidade clínica do texto para análise ou treinamento."},


'Re-identification': {'score': 9,
                      'text': "O risco de reidentificação humana da paciente com base no prontuário anonimizado pelo modelo é baixo.
                               As principais informações diretamente identificáveis (nome do profissional de saúde, seu número de Coren,
                               e a data exata do atendimento) foram eficazmente removidas e substituídas por tags. As informações clínicas restantes,
                               como o procedimento 'broncoscopia', observações sobre acessos venosos periféricos (AVP em MSD/MSE), e os escores de avaliação,
                               são comuns em contextos hospitalares e, na ausência de identificadores diretos como nome do hospital, cidade,
                               ou uma data precisa (substituída por '<DATE>'), dificilmente permitiriam a reidentificação unívoca de um indivíduo.
                               A manutenção do horário ('15:46'), associado a uma '<DATE>' genérica, não constitui um risco significativo por si só.
                               Não foram identificadas combinações de informações quasi-identificadoras residuais que representassem um risco elevado.
                               Para que a reidentificação ocorresse, seria necessário acesso a um conjunto de dados externo muito específico e restrito,
                               o que torna o risco baixo, mas não absolutamente nulo, justificando a nota 9."}}
```


**Figure 6.** Sample evaluations generated by Gemini 2.5 Pro analyzing the tuple containing the original medical record, a version anonymized by a human annotator, and a version anonymized by a language model, based on the three evaluation criteria from the LLM-as-a-Judge section: Technical Performance, Information Loss, and Re-identification Risk.

```
{'Performance': {'score': 10,
                 'text': "The language model demonstrated perfect technical performance in anonymization, exactly replicating the specialist's result.
                         All identifiable information present in the original medical record was correctly removed or masked.
                         Specifically, the nurse's name ('Gabriela Cardoso') was replaced with the generic tag '<DOCTOR> <DOCTOR>',
                         the Coren number ('329999') was replaced with '<IDNUM>', and the full date ('17/02/2017') was replaced with '<DATE>'.
                         The time ('15:46') was retained, which is consistent with the specialist's anonymization and, in the absence of the full date,
                         represents a very low re-identification risk. There were no other direct PII in the body of the text requiring removal,
                         and the model did not introduce any false positives. The only minimal alteration involved some extra spaces around punctuation and tags,
                         such as 'Enf . <DOCTOR> <DOCTOR> , Coren <IDNUM> em <DATE> 15:46', which do not affect the quality of the anonymization itself."},


'Loss': {'score': 9,
         'text': "The anonymization performed by the model preserved almost entirely the useful information for clinical analysis and
                 deep learning model training. The main body of the medical record, containing clinical observations about the patient's condition,
                 procedures ('bronchoscopy'), physical exams, and scores (FUGULIN, Phlebitis, MORSE, BRADEN), remained unaltered and readable,
                 maintaining its semantic value. Replacing the professional's information with generic tags is a standard practice that removes PII
                 without compromising the clinically relevant content. The slight deduction in score (from 10 to 9) is due to a typographical/concatenation error
                 ('lesões depele' instead of 'lesões de pele') introduced by the model and slightly abnormal spacing in the signature line
                 ('Enf . <DOCTOR> <DOCTOR> , Coren <IDNUM> em <DATE> 15:46'). Although 'Enf.' was replaced with '<DOCTOR> <DOCTOR>',
                 the professional category 'Enf.' was preserved, which is positive. These are minor issues that do not substantially affect
                 the clinical utility of the text for analysis or training."},


'Re-identification': {'score': 9,
                      'text': "The human re-identification risk of the patient based on the medical record anonymized by the model is low.
                              The main directly identifiable information (healthcare professional's name, Coren number,
                              and exact date of service) was effectively removed and replaced with tags. The remaining clinical information,
                              such as the 'bronchoscopy' procedure, observations on peripheral venous access (AVP in MSD/MSE), and evaluation scores,
                              are common in hospital contexts and, in the absence of direct identifiers such as hospital name, city,
                              or a precise date (replaced with '<DATE>'), would hardly allow the unique re-identification of an individual.
                              Retaining the time ('15:46'), associated with a generic '<DATE>', does not constitute a significant risk on its own.
                              No residual quasi-identifiable information combinations were identified that would represent a high risk.
                              For re-identification to occur, access to a very specific and restricted external dataset would be required,
                              which makes the risk low but not absolutely null, justifying the score of 9."}}
```

**Figure 7.** Translated sample evaluations generated by Gemini 2.5 Pro analyzing the tuple containing the original medical record, a version anonymized by a human annotator, and a version anonymized by a language model, based on the three evaluation criteria from the LLM-as-a-Judge section: Technical Performance, Information Loss, and Re-identification Risk.

## LLM-as-a-Judge Reports Prompt

In this section, we present the prompt used to guide the Gemini 2.5 Pro model to generate reports using the results of the LLM-as-a-Judge evaluation. Figure 8 shows the prompt in Portuguese, while Figure 9 displays the corresponding English translation. The prompt contains detailed instructions for analyzing a list of anonymized medical records, specifying the evaluation of one of three criteria: Technical Anonymization Performance, Information Loss, or Human Re-identification Risk. It also provides guidelines for generating a synthesized report that identifies general trends, recurring strengths and weaknesses, and main issues, with an output format of one or two paragraphs up to 300 words.

```
Você é um especialista em privacidade de dados médicos e anonimização de textos clínicos.
Sua tarefa é analisar uma lista de avaliações sobre prontuários médicos que foram anonimizados por um modelo de linguagem.

Cada entrada da lista representa a avaliação de um prontuário que leva em consideração um desses três aspectos:

1. **Desempenho técnico da anonimização** - quão bem as informações sensíveis foram removidas ou mascaradas.
2. **Perda de informação** - o quanto a utilidade clínica do texto foi preservada após a anonimização.
3. **Risco de reidentificação humana** - a possibilidade de que um humano consiga identificar o paciente com base nas informações restantes.

Sua tarefa é gerar um relatório que:

- Identifique **tendências gerais** nas avaliações para o critério especificado.
- Destaque **pontos fortes e fracos recorrentes** destacados nas avaliações para o critério especificado.
- Indique os principais problemas destacados nas avaliações para o critério especificado.

No início do texto estará indicado qual critério deverá ser avaliado.

Formato da saída:
- Um parágrafo ou dois com até 300 palavras.
- Não repita todos os diagnósticos, mas **sintetize os achados globais**.

Diagnósticos de entrada:
<<INSIRA AQUI A LISTA DE AVALIAÇÕES GERADAS>>
```

**Figure 8.** Prompt used to guide the Gemini 2.5 Pro model in generating reports based on the results of the LLM-as-a-Judge evaluation.

```
You are a specialist in medical data privacy and clinical text anonymization.
Your task is to analyze a list of evaluations of medical records that were anonymized by a language model.

Each entry in the list represents the evaluation of a medical record considering one of the following aspects:

1. **Technical anonymization performance** - how well sensitive information was removed or masked.
2. **Information loss** - the extent to which the clinical utility of the text was preserved after anonymization.
3. **Human re-identification risk** - the likelihood that a human could identify the patient based on the remaining information.

Your task is to generate a report that:

- Identifies general trends in the evaluations for the specified criterion.
- Highlights recurring strengths and weaknesses noted in the evaluations for the specified criterion.
- Indicates the main issues highlighted in the evaluations for the specified criterion.

At the beginning of the text, the criterion to be evaluated will be indicated.

Output format:
- One or two paragraphs, up to 300 words.
- Do not repeat all individual diagnoses, but **synthesize the overall findings**.

Input evaluations:
<<INSERT THE LIST OF GENERATED EVALUATIONS HERE>>
```

**Figure 9.** English translation of the prompt used to guide the Gemini 2.5 Pro model in generating reports based on the results of the LLM-as-a-Judge evaluation.

## LLM-as-a-Judge Reports

In this section, we present sample reports generated by Gemini 2.5 Pro based on the three evaluation criteria defined in the LLM-as-a-Judge framework: Technical Performance, Information Loss, and Re-identification Risk. These reports are produced by the model after processing multiple anonymization examples, such as those illustrated in the previous section. Figure 10 displays the reports in Portuguese, and Figure 11 presents their English translations.

- Technical Performance
As avaliações sobre o desempenho técnico da anonimização indicam uma capacidade geralmente boa do modelo em lidar com identificadores diretos e comuns.
Frequentemente, o modelo remove com sucesso a maioria das informações sensíveis. Pontos fortes recorrentes incluem alta precisão no mascaramento de nomes
completos de pacientes e médicos, números de CRM, idades (quando explicitamente declaradas) e datas em formatos padronizados (ex: DD/MM/AAAA),
utilizando tags como \`<PATIENT>\`, \`<DOCTOR>\`, \`<IDNUM>\`, \`<AGE>\` e \`<DATE>\`.
Muitas avaliações destacam um desempenho "perfeito" ou "muito bom" nestes aspectos, equiparando-se, por vezes, à anonimização feita por especialistas humanos.
No entanto, surgem fraquezas e problemas significativos. Uma tendência preocupante é a dificuldade em lidar com datas parciais,
durações (frequentemente confundidas com \`<AGE>\` ou não anonimizadas) e horários. A anonimização de localizações (cidades, estados, instituições) é outro ponto fraco,
com erros de classificação (ex: cidade como profissão) e remoção incompleta de nomes compostos. Informações quasi-identificadoras como profissões, estado civil,
escolaridade e religião são frequentemente omitidas ou mal classificadas.
Falhas críticas incluem a não remoção de sobrenomes de médicos em alguns contextos e, de forma mais alarmante, a corrupção do texto original.
Esta última manifesta-se pela inserção de dados ou tags incorretas, duplicação de trechos ou exclusão de informações clínicas relevantes adjacentes à PII processada,
sendo um dos principais problemas destacados.

- Information Loss
As avaliações sobre a perda de informação indicam uma tendência geral de que o modelo, em muitos casos, consegue preservar adequadamente a informação clínica essencial
quando as informações identificáveis são substituídas por tags genéricas. Nesses cenários positivos, diagnósticos, históricos médicos, medicações,
achados de exames físicos e complementares, e a narrativa clínica são mantidos, resultando em perda mínima de utilidade para análise clínica ou treinamento de modelos.
A legibilidade e o valor semântico são frequentemente preservados, permitindo a compreensão do caso clínico.
No entanto, os pontos fracos e problemas recorrentes emergem consistentemente quando o modelo comete erros de classificação ou interpretação.
A principal fonte de perda de informação significativa ocorre devido à corrupção de dados clínicos, como a alteração de termos diagnósticos (ex: "Granangeite"),
a substituição de informações clínicas por tags incorretas ou dados descontextualizados (ex: sintomas urêmicos substituídos por dados microbiológicos,
"TEC < 3 seg" por "<DATE>"), e a má interpretação semântica de informações contextuais (ex: "dois filhos" como "<PROFESSION>", "Paulínia-SP" como "<PROFESSION>").
Outro problema relevante é a omissão de informações clinicamente importantes, como frases sobre o estado do paciente ("dor controlada com dipirona"),
exposições relevantes ("exposição a fogão a lenha") ou até seções de exames. Finalmente, a duplicação de trechos e a introdução de erros que quebram a coerência textual
(ex: "após" tornado "<DATE>") também são citados como fatores que diminuem a fidelidade e utilidade dos prontuários anonimizados.

- Re-identification Risk
As avaliações sobre o risco de reidentificação humana indicam uma tendência de risco frequentemente classificado entre moderado e considerável.
Um ponto forte recorrente do modelo é a remoção de identificadores diretos, como nomes de pacientes e datas completas, que são geralmente substituídos por tags genéricas.
No entanto, os pontos fracos são significativos e frequentes, com destaque para a falha em anonimizar ou mascarar adequadamente quasi-identificadores.
Os principais problemas identificados incluem a manutenção de profissões específicas ("limpeza em loja de peça de carros", "padaria"),
nomes de profissionais de saúde ("Pires", "Dr Gustavo Franco"), fragmentos de datas ou horários ("D0 20-10", "agosto/23", "18h"),
e localizações geográficas parciais ("Ribeirão"). Adicionalmente, muitas avaliações ressaltam que a combinação de múltiplos detalhes clínicos
altamente específicos ou raros (como politraumas com causas incomuns, conjuntos de diagnósticos complexos e raros, ou históricos sociais distintivos),
mesmo que os identificadores diretos tenham sido removidos, pode criar um "efeito mosaico", permitindo a reidentificação por indivíduos com conhecimento contextual ou
acesso a informações complementares. Falhas pontuais na categorização de tags, como classificar "divorciado" como profissão, também foram notadas,
embora o impacto maior venha da exposição de dados sensíveis.

**Figure 10.** Sample reports generated by Gemini 2.5 Pro based on the three evaluation criteria from the LLM-as-a-Judge section: Technical Performance, Information Loss, and Re-identification Risk.

- Technical Performance
Evaluations of the technical performance of anonymization indicate a generally good ability of the model to handle direct and common identifiers.
The model frequently succeeds in removing most sensitive information. Recurring strengths include high precision in masking full names of patients and doctors,
CRM numbers, ages (when explicitly stated), and dates in standard formats (e.g., DD/MM/YYYY), using tags such as <PATIENT>, <DOCTOR>, <IDNUM>, <AGE>, and <DATE>.
Many evaluations highlight a “perfect” or “very good” performance in these aspects, sometimes equating to the anonymization performed by human specialists.
However, significant weaknesses and issues emerge. A concerning trend is the difficulty in handling partial dates,
durations (often confused with <AGE> or left unanonymized), and time expressions. The anonymization of locations (cities, states, institutions) is another weak point,
with classification errors (e.g., city as profession) and incomplete removal of compound names. Quasi-identifiers such as profession, marital status,
education level, and religion are often omitted or misclassified.
Critical failures include the non-removal of doctors' surnames in some contexts and, more alarmingly, corruption of the original text.
This is manifested by the insertion of incorrect tags or data, duplication of passages, or deletion of clinically relevant information adjacent to the processed PII,
being one of the main issues reported.

- Information Loss
Evaluations of information loss indicate a general trend in which the model often manages to adequately preserve essential clinical information
when identifiable elements are replaced by generic tags. In these positive scenarios, diagnoses, medical history, medications,
findings from physical and complementary exams, and clinical narratives are maintained, resulting in minimal loss of utility for clinical analysis or model training.
Readability and semantic value are frequently preserved, allowing understanding of the clinical case.
However, consistent weaknesses and problems emerge when the model misclassifies or misinterprets entities.
The main source of significant information loss stems from corruption of clinical data, such as altering diagnostic terms (e.g., "Granangeite"),
replacing clinical information with incorrect tags or decontextualized data (e.g., uremic symptoms replaced by microbiological data,
"TEC < 3 seg" replaced by <DATE>), and semantically misinterpreting contextual information (e.g., "two children" as <PROFESSION>, "Paulínia-SP" as <PROFESSION>).
Another relevant issue is the omission of clinically important information, such as statements about the patient's condition ("pain controlled with dipyrone"),
relevant exposures ("exposure to wood stove smoke"), or even exam sections. Lastly, duplication of passages and the introduction of coherence-breaking errors
(e.g., "after" converted to <DATE>) are also noted as factors that reduce the fidelity and usefulness of the anonymized records.

- Re-identification Risk
Evaluations of residual re-identification risk indicate a trend of moderate to considerable risk.
A recurring strength of the model is the removal of direct identifiers, such as patient names and full dates, which are generally replaced with generic tags.
However, weaknesses are significant and frequent, especially regarding the failure to adequately anonymize or mask quasi-identifiers.
Major issues include the retention of specific professions ("cleaning in auto parts store", "bakery"),
healthcare professional names ("Pires", "Dr Gustavo Franco"), date or time fragments ("D0 20-10", "August/23", "6 p.m."),
and partial geographic locations ("Ribeirão"). Additionally, many evaluations emphasize that the combination of multiple highly specific or rare clinical details
(such as polytraumas with unusual causes, sets of complex and rare diagnoses, or distinctive social histories),
even when direct identifiers have been removed, can create a "mosaic effect", enabling re-identification by individuals with contextual knowledge or
access to complementary information. Occasional failures in tag categorization, such as labeling "divorced" as a profession, were also noted,
though the greatest impact results from the exposure of sensitive data.

**Figure 11.** Translated sample reports generated by Gemini 2.5 Pro based on the three evaluation criteria from the LLM-as-a-Judge section: Technical Performance, Information Loss, and Re-identification Risk.
